# Adding Weekly Intramuscular Progesterone to a Twice Daily Vaginal Progesterone Capsule for Luteal Phase Support in IVF/ICSI Cycles Results in Similar Live Birth Rates

**DOI:** 10.5935/1518-0557.20210040

**Published:** 2022

**Authors:** Salwa Gari, Dania Al-Jaroudi

**Affiliations:** 1 Reproductive Endocrinology and Infertility Fellow, Reproductive Endocrinology & Infertility Medicine Department, Obstetrics and Gynecology, Women's Specialized Hospital King Fahad Medical City, Riyadh, Saudi Arabia; 2 Reproductive Endocrinology and Infertility Consultant, Minimally Invasive Gynecologic Surgery, Reproductive Endocrinology & Infertility Medicine Department, Obstetrics and Gynecology, King Fahad Medical City, Kingdom of Saudi Arabia

**Keywords:** vaginal progesterone, intramuscular progesterone, ART, IVF, luteal phase support, live birth

## Abstract

**Objective:**

To compare pregnancy outcomes in patients undergoing artificial reproductive treatment (ART) and fresh embryo transfer (ET) who received twice-daily vaginal progesterone capsule (Cyclogest) alone verses twice daily vaginal progesterone capsule (Cyclogest) plus weekly intramuscular Hydroxyprogesterone Capronate (Proluton depot) for luteal phase support.

**Methods:**

A retrospective cohort study that included 1162 patients who completed fresh ART/ET cycle from January 2015 to April 2018. Vaginal Cyclogest 400 mg twice daily was given to 985 patients following oocytes retrieval; whereas 177 patients received weekly intramuscular Proluton depot 250 mg in addition to twice-daily Cyclogest. The primary outcome was live birth rate. The secondary outcomes included biochemical pregnancy rate, clinical pregnancy, biochemical, and early and late pregnancy loss.

**Results:**

There was no difference between the twice-daily vaginal progesterone and the addition of weekly intramuscular progesterone injections to the twice-daily vaginal progesterone regarding a positive pregnancy test (40.5% and 46.9%, respectively, *p*=0.112). There was no statistical difference in live birth rates between the groups (24% for group one, 26% for group two, *p*=0.582).

**Conclusions:**

The administration of weekly intramuscular progesterone in addition to twice-daily vaginal progesterone capsule for luteal phase support post ART cycle does not result in higher live birth rate.

## INTRODUCTION

During the luteal phase, the corpus luteum produces progesterone, which prepares the estrogen-primed endometrium for embryo implantation (Heine *et al*., 2017). Progesterone is known to be an essential hormone required for successful implantation and continuation of early pregnancy by inducing sequential events in the endometrium during the luteal phase, including maturation, secretory transformation of the glandular elements, edema formation, vascular proliferation and stroma decidualization (Heine *et al*., 2017; Ho *et al*., 2008; Satir *et al*., 2013; Beltsos *et al*., 2014). In assisted reproductive technology (ART) treatment, the down regulation of luteinizing hormone (LH) secretion by the use of gonadotropin-releasing hormone (GnRH) agonists or antagonists, can lead to luteal phase disruption as the amount of progesterone produced is not sufficient; this can lead to ineffective development of the endometrium, hence reduction in the implantation rate (Heine *et al*., 2017; Ho *et al*., 2008; Satir *et al*., 2013; Beltsos *et al*., 2014; Bahceci & Ulug, 2008).

Luteal phase support following ovarian super-stimulation and oocyte retrieval had been the focus of many researches since the beginning of ART (Bahceci & Ulug, 2008). Progesterone is critical in achieving endometrial- embryo synchrony and maintaining the early pregnancy until the placenta becomes fully developed, around the tenth or twelfth weeks of pregnancy (Shapiro *et al*., 2014).

During in vitro fertilization (IVF), the women take either progesterone, human chorionic gonadotropin (hCG) or GnRH agonist medication for luteal support; progesterone is the most commonly used. There are different routes for progesterone administration, oral, vaginal, rectal, intramuscular, with all showing similar levels of efficacy, except for the oral progesterone administration, due to its poor bioavailability. Side effects from intramuscular progesterone are pain at the injection site, and local abscess; side effects from the vaginal progesterone are local irritation, vaginal discharge, and vaginal bleeding (Barbosa *et al*., 2018).

There is no consensus as to which is the best luteal phase support regimen (Tay & Lenton, 2005). Progesterone is typically given from the day of oocyte retrieval until the tenth or twelfth week of pregnancy, each route of administration has different pharmacokinetics, potency and adverse effects (Kokeguchi *et al*., 2016).

Although luteal phase support following IVF/intracytoplasmic sperm injection (ICSI) cycles has been extensively studied throughout the years of ART, a standard protocol for luteal phase support has not been established (i.e. optimal formula, dose, route, timing and duration), and the comparison between vaginal and intramuscular progesterone have yielded conflicting results (Wang *et al*., 2009; Khrouf *et al*., 2017; Gün *et al*., 2016; Sofuoglu *et al*., 2015). The objective of this study was to compare live birth rates (LBR), implantation rates (IR) and clinical pregnancy rates (CPR) after IVF/ICSI cycles in patients receiving either vaginal progesterone alone or vaginal progesterone along with intramuscular (IMP) progesterone.

## MATERIAL AND METHODS

We ran a retrospective cohort study, in which we analyzed the medical records of patients seen at the Reproductive Endocrine and Infertility Medicine Department (REIMD) at King Fahad Medical City, Riyadh between January 2015 and April 2018. The institutional review board approved the study (18-530). Patients below the age of 18 years or above 40 years of age, with body mass index (BMI) below 18 or above 40 kg/m^2^, history of ≥ three miscarriages, patients with natural cycles, patients with cycle cancellation before ovum pick up or patients who failed to reach embryo transfer stage, patients who had freeze-all cycles due to risk of ovarian hyper-stimulation syndrome (OHSS), or any other cause and any cycle that was converted to intrauterine insemination (IUI) were excluded.

There were one thousand and sixty-two treatment cycles in the review. All the patients were nulliparous. We obtained demographic characteristics, causes of infertility, related obstetric history, stimulation protocols, doses of medications for stimulation and luteal support and treatment outcomes from patients' files. The patients were divided randomly into two groups; those who received vaginal progesterone (VP) (Cyclogest 400 mg, Actavis, Barnstaple, UK) for luteal support and those who received vaginal progesterone (Cyclogest 400 mg, Actavis, Barnstaple, UK) along with intramuscular (IMP) Hydroxyprogesterone Caproate (IMP) (Proluton depot 250 mg, Bayer Pharma AG, Germany).The continuous variables were categorized into ordinal and nominal groups, and summarized using proportions.

All the patients underwent complete infertility evaluation, including early follicular phase serum follicle-stimulating hormone (FSH), luteinizing hormone (LH), and estradiol (E2) level measurements, and a baseline transvaginal ultrasound examination (TVUS) during their second or third menstrual period. The patients were randomly allocated to either long or antagonist protocol. In the long agonist group, the patients received 1.88 mg depot leuprolide acetate (Lupron, Abbott Laboratories, Chicago, IL) intramuscularly on day 21 of the cycle. Following 12 to 15 days after Lupron, absence of ovarian follicles, endometrial thickness ≤ six mm on TVUS examination, and plasma E2 levels ≤ 250 mmol/L were checked for desensitization. Once verified, the patients began treatment with either daily injection of either recombinant FSH (GONAL- f, Merck Serono, Darmstadt, Germany) or human menopausal gonadotropin (Merional, IBSA, Lugano, Switzerland) or r-FSH in addition to r-LH (Lutropin alfa, Merck Serono, Darmstadt, Germany). The initial gonadotropin dose was individualized according to the patient's age, BMI, ovarian reserve determined by antral follicle count and basal FSH level, and the response of a previous cycle. We adjusted the gonadotropin doses after five or six days of stimulation based on E2 levels and TVUS; when three follicles reached a mean diameter ≥ 17 mm, patients received 10,000 IU IM human chorionic gonadotrophin (hCG) (Pregnyl, Merck, Kenilworth, NJ) for final oocyte maturation.

As for the GnRH antagonist protocol group, daily injections of gonadotropins commenced on the second or third day of menstrual period with varying individualized doses, as described earlier. Daily subcutaneous injections of 0.25 mg GnRH antagonist (Cetrotide, Serono, Geneva, Switzerland) was started on day five or six of the stimulation, or when the leading follicle was 13 to 14 mm in diameter and then continued daily until hCG injection was given.

TVUS-guided oocyte retrieval was performed 36 hours after hCG administration. Retrieved oocytes were inseminated with sperm using IVF if the semen analysis was normal. ICSI was performed for severe male factor infertility or in patients experiencing previously failed fertilization after IVF or unexplained infertility. We assessed fertilization 16-18 hours after insemination and transfer of one to two fresh embryos on days two to five after fertilization into the uterine cavity, performed under ultrasound guidance. The patients were randomly categorized into two groups according to the luteal support treatment, which was commenced on the day following oocyte retrieval. In the first group, the patients were prescribed 400 mg of vaginal progesterone (VP) pessary (Cyclogest, Actavis, Barnstaple, UK) twice daily. The second group were given 400 mg VP twice daily plus weekly intramuscular Hydroxyprogesterone Capronate (Proluton depot 250 mg, Bayer Pharma AG, Germany), which was continued if pregnancy was achieved for 12 weeks. Unfortunately, mid-luteal progesterone level was not measured due to lack of availability in our hospital.

Biochemical pregnancy (BPR) was defined as a positive pregnancy test result (β-hCG levels > 10 mIU/ml) 12 days after embryo transfer. Clinical pregnancy rate (CPR) was defined as the presence of a gestational sac seen by vaginal ultrasonography four or five weeks after oocyte retrieval, the biochemical pregnancy loss was the positive pregnancy test associated with a low concentration of serum β-hCG, followed by rapid fall and possible delay in menstruation, late pregnancy loss was the loss of pregnancy between 13 weeks till ≥ 23+6 weeks, and live birth rate (LBR) was defined as number of deliveries that resulted in live born neonate after 24 weeks of gestation.

### Statistical analysis

All Categorical variables presented as numbers and percentages. Continuous variables were expressed as Mean ± SD. Chi-square/Fisher's exact test was used according to whether the cell expected frequency is smaller than five. Independent sample t-tests applied to determine the mean significant differences between the groups. We used the Mann-Whitney test to find out median differences. A p-value lower than 0.05 was considered statistically significant. All data was entered and analyzed through the SPSS 25 statistical package (SPSS Inc., Chicago, IL, USA).

## RESULTS

We reviewed one thousand thirty-two records. Four hundred and sixty-one records were excluded because they did not fulfill the inclusion criteria. One thousand and sixty-two patients' charts were analyzed. Nine hundred and eighty-five patients in group one received Cyclogest twice daily and 177 patients in group two received weekly Proluton injection in addition to the twice-daily Cyclogest. There was no statistical difference with respect to the demographic data or obstetric history, the mean age of group one was 31.13±4.86 years versus group two 31.72±5.04 years (*p*=0.143), the BMI of group one was 28.32±4.25kg/m2 while for group two it was 28.37±4.26kg/m^2^ (*p*=0.888) (Table 1). There was no difference in the rate of miscarriage in both groups. There was a statistical difference between both groups in terms of previous ectopic pregnancy 15 (1.5%) in group one, versus seven (4.0%) in group two (*p*=0.029) (Table 1).

**Table 1:** Basic demographic characteristics and past obstetric history differences of the two groups.

		Luteal phase support		*p*-*value*
		Cyclogest (n=985)	Cyclogest/Proluton (n=177)	
Age	Mean ± SD	31.13±4.86	31.72±5.04	0.143
BMI	Mean ± SD	28.32±4.25	28.37±4.26	0.888
Miscarriage	No miscarriage	864 (87.7%)	147 (83.1%)	0.089
	Less than 3 miscarriage	121 (12.3%)	30 (16.9%)	
Ectopic pregnancy	No ectopic pregnancy	970 (98.5%)	170 (96.0%)	0.029
	more than one ectopic pregnancy	15 (1.5%)	7 (4.0%)	
Menses	Regular	783 (79.5%)	129 (72.9%)	0.049
	Irregular	193 (19.6%)	45 (25.4%)	0.077
	Amenorrhea	9 (0.9%)	3 (1.7%)	0.344

The mean duration of infertility was 5.97±3.50 years in group one while in group two it was 6.65±3.92 years (*p*=0.031), the main cause of infertility was male factor in both groups. Three hundred and ten (31.5%) in the VP group and 55 (31.1%) in VP+ IMP (*p*=0.916) followed by unexplained infertility 254 (25.8%) in group one and 48 (27.1%) in-group 2, *p*=0.710.

There was no statistical difference between stimulation protocols in the two groups (Table 2). Nine hundred and forty-one (95.5%) had the ICSI procedure while 44 (4.5%) had the IVF procedure in Group one, and 172 (97.2%) vs. five (2.8%) in Group two, respectively, *p*=0.317. Most of the cases had two embryos transferred. The duration of ovarian stimulation, the total dose of gonadotropins and the stimulation duration were comparable between the two groups (Table 2). The number of oocytes retrieved, number of embryos generated did not differ between the groups. Most of the patients had embryo transfer on day three (Table 2).

**Table 2:** Stimulation cycle characteristics in both groups.

		Luteal phase support		*p - value*
		Cyclogest (n=985)	Cyclogest/Proluton (n=177)	
Protocol	Antagonist	734 (74.5%)	131 (74.0%)	0.887
	Long	251 (25.5%)	46 (26.0%)	
Procedure	ICSI	941 (95.5%)	172 (97.2%)	0.317
	IVF	44 (4.5%)	5 (2.8%)	
Total gonadotropin Dose	Median (IQR)	1800 (2700 - 1350)	2025 (3225 - 1225)	0.196
Number of eggs	Median (IQR)	7 (10 - 5)	7 (10 - 4.5)	0.693
Number of embryos generated	Median (IQR)	4 (6 - 2)	4 (6 - 2)	0.787
Number of embryos transferred	1	169 (17.2%)	35 (19.8%)	0.400
	2	795 (80.7%)	140 (79.1%)	0.618
	3	21 (2.1%)	2 (1.1%)	0.378
ET Day	2	287 (29.1%)	54 (30.5%)	0.712
	3	582 (59.1%)	107 (60.5%)	0.733
	4	48 (4.9%)	9 (5.1%)	0.904
	5	68 (6.9%)	7 (4.0%)	0.142
FSH drug	Recombinant FSH (Gonal-F)	528 (53.6%)	86 (48.6%)	0.218
	Human menopausal gonadotropin (Merional)	397 (40.3%)	74 (41.8%)	0.708
	Recombinant FSH (Gonal-F) + Human menopausal gonadotropin (Merional)	48 (4.9%)	14 (7.9%)	0.098
	Recombinant FSH (Gonal-F) + recombinant LH (Lutropin alfa)	12 (1.2%)	3 (1.7%)	0.605

The biochemical pregnancy rates were 399 (40.5%) and 83 (46.9%) (*p*=0.112), respectively. BPR, early and late pregnancy loss was the same in both the groups (Table 3).

**Table 3:** Pregnancy outcomes of IVF/ICSI cycles.

		Luteal phase support		*p* - value
		Cyclogest (n=985)	Cyclogest/Proluton (n=177)	
Pregnancy test	Positive	399 (40.5%)	83 (46.9%)	0.112
	Negative	586 (59.5%)	94 (53.1)	
Outcomes	Single	192 (48.0%)	37 (44.6%)	0.664
	Twin	44 (11.0%)	8 (9.6%)	<0.001
	More than 2 babies	1 (0.3%)	1 (1.2%)	0.171
	Abortion	161 (40.3%)	35 (42.2%)	0.348
	Ectopic	2 (0.5%)	3 (3.6%)	0.005
Delivery	Normal vaginal delivery	91 (22.8%)	15 (16.9%)	0.570
	Caesarean section	146 (36.5%)	31 (37.3%)	0.359
	Abortion	161 (40.3%)	35 (42.2%)	0.262
	Ectopic	2 (0.5%)	3 (3.6%)	0.005
Gestational age	More than or equal 34 weeks	208 (52.0%)	37 (44.6%)	0.949
	Between 24-34 weeks	27 (6.8%)	8 (9.6%)	0.202
	Late fetal loss	4 (1.0%)	2 (2.4%)	0.216
	Early fetal loss	130 (32.5.0%)	27 (32.5%)	0.461
	Biochemical loss	29 (7.2%)	6 (7.2%)	0.749
	Ectopic	2 (0.5%)	3 (3.6%)	0.005
Live birth rate		237 (24.0%)	46 (26.0%)	0.582

The live birth rates were similar in both groups; 233 (24%) for group one, and 45 (26%) for group two (*p*=0.582). Table 3 illustrates pregnancy outcomes from IVF/ICSI cycles.

## DISCUSSION

Our study has demonstrated that the addition of IM progesterone to vaginal progesterone does not show any improvement to pregnancy and live birth rates. There was no significant difference in pregnancy outcomes regarding clinical pregnancies, miscarriage and live births between the vaginal progesterone group and the VP group with intramuscular progesterone. It is worth mentioning that despite the fact that there was no statistical difference between the two groups in terms of pregnancy rate, 40.5% in-group one verses 46.9% in group two, the percentages were higher in the second group. However, this should be interpreted with caution since the numbers in both groups are different and an estimate of statistical power associated with the various outcomes was not determined due to the retrospective nature of our study. Additionally, there were similar live birth between both groups, 237 (24%) in the Cyclogest group and 46 (26%) in the Cyclogest and the IMP progesterone group. Progesterone has been used traditionally for luteal phase support in cycles of assisted reproduction, and resulted in higher live birth rates in comparison to placebo (Gün *et al*., 2016; Jabara *et al*., 2009; Doblinger *et al*., 2016; Michnova *et al*., 2017), and shows less ovarian hyper-stimulation as compared to hCG for luteal phase support (Baker *et al*., 2014; Zaman *et al*., 2017).

Our study results are similar to a retrospective cohort that compared both pregnancy rates and LBR after an IVF-ET in patients who used either vaginal micronized progesterone alone or those who used it along with Progesterone in the oil injection, and VP for luteal phase support (Abuzeid *et al*., 2015). It is also consistent with other studies that found no statistical clinical differences between VP and IMP in terms of patient preference, drug efficacy, safety IR, CPR, and LBR (Shapiro *et al*., 2014; Zaman *et al*., 2017; Tournaye *et al*., 2017).

One retrospective study suggested that the route of luteal phase support could be a significant prognostic factor in relation to the stimulation protocol. Bahceci & Ulug (2008) found higher BPR, but lower IR in the VP group, compared to the IMP group in the antagonist protocol only. Our results are consistent with their findings in terms of the same CPR and LB among the agonist group. However, we did not detect any difference between agonist and antagonist groups with respect to BPR and LBR. Two other studies analyzed VP in comparison to IMP for luteal phase support and found that VP resulted in better clinical pregnancy outcomes that could be due to progesterone's local effect inducing secretory transformation of the endometrial stroma (Ho *et al*., 2008; Satir *et al*., 2013; Asoglu *et al*., 2019). This study is limited due to its retrospective design. Therefore, in clinical practice, before considering vaginal progesterone along with intramuscular progesterone as a choice for luteal phase support, further randomized clinical trials should be conducted to support such recommendation.

In conclusion, the administration of weekly IMP in addition to twice daily VP capsule for luteal phase support post ART cycle does not result in higher live birth rates; however, more randomized clinical trials are still warranted to assess the optimal route of luteal supplementation to enhance live birth rates.

## References

[r1] Abuzeid O, Hebert J, Abozaid TI, Ashraf M, Abuzeid MI (2015). Comparison of vaginal micronized progesterone and combined intramuscular and vaginal progesterone for luteal phase support during in vitro fertilization - embryo transfer treatment in women < 35 years. Fertil Steril.

[r2] Asoglu MR, Celik C, Karakis LS, Findikli N, Gultomruk M, Bahceci M (2019). Comparison of daily vaginal progesterone gel plus weekly intramuscular progesterone with daily intramuscular progesterone for luteal phase support in single, autologous euploid frozen-thawed embryo transfers. J Assist Reprod Genet.

[r3] Baker VL, Jones CA, Doody K, Foulk R, Yee B, Adamson GD, Cometti B, DeVane G, Hubert G, Trevisan S, Hoehler F, Jones C, Soules M (2014). A randomized, controlled trial comparing the efficacy and safety of aqueous subcutaneous progesterone with vaginal progesterone for luteal phase support of in vitro fertilization. Hum Reprod.

[r4] Bahceci M, Ulug U (2008). Route of progesterone administration for luteal phase support may affect outcome of controlled ovarian hyperstimulation for IVF with ICSI using GnRH antagonist. J Assist Reprod Genet.

[r5] Barbosa MWP, Valadares NPB, Barbosa ACP, Amaral AS, Iglesias JR, Nastri CO, Martins WP, Nakagawa HM (2018). Oral dydrogesterone vs. vaginal progesterone capsules for luteal-phase support in women undergoing embryo transfer: a systematic review and meta-analysis. JBRA Assist Reprod.

[r6] Beltsos AN, Sanchez MD, Doody KJ, Bush MR, Domar AD, Collins MG (2014). Patients' administration preferences: progesterone vaginal insert (Endometrin®) compared to intramuscular progesterone for Luteal phase support. Reprod Health.

[r7] Doblinger J, Cometti B, Trevisan S, Griesinger G (2016). Subcutaneous Progesterone Is Effective and Safe for Luteal Phase Support in IVF: An Individual Patient Data Meta-Analysis of the Phase III Trials. PLoS One.

[r8] Gün İ, Özdamar Ö, Şahin S, Çetingöz E, Sofuoğlu K (2016). Progesterone vaginal capsule versus vaginal gel for luteal support in normoresponder women undergoing long agonist IVF/ICSI cycles. Ginekol Pol.

[r9] Heine P, Sellar L, Whitten S, Bajaj P (2017). A questionnaire-based audit to assess overall experience and convenience among patients using vaginal progesterone tablets (Lutigest®) for luteal phase support during IVF treatment. Patient Relat Outcome Meas.

[r10] Ho CH, Chen SU, Peng FS, Chang CY, Yang YS (2008). Luteal support for IVF/ICSI cycles with Crinone 8% (90 mg) twice daily results in higher pregnancy rates than with intramuscular progesterone. J Chin Med Assoc.

[r11] Jabara S, Barnhart K, Schertz JC, Patrizio P (2009). Luteal phase bleeding after IVF cycles: comparison between progesterone vaginal gel and intramuscular progesterone and correlation with pregnancy outcomes. J Exp Clin Assist Reprod.

[r12] Khrouf M, Slimani S, Khrouf MR, Braham M, Bouyahia M, Berjeb KK, Chaabane HE, Merdassi G, Kaffel AZ, Zhioua A, Zhioua F (2017). Progesterone for Luteal Phase Support in In Vitro Fertilization: Comparison of Vaginal and Rectal Pessaries to Vaginal Capsules: A Randomized Controlled Study. Clin Med Insights Womens Health.

[r13] Kokeguchi S, Hayashi N, Rogoff D, Shimizu S, Ishihara O (2016). Phase III trial of 8% vaginal progesterone gel for luteal phase support in Japanese women undergoing in vitro fertilization and fresh embryo transfer cycles. Reprod Med Biol.

[r14] Michnova L, Dostal J, Kudela M, Hamal P, Langova K (2017). Vaginal use of micronized progesterone for luteal support. A randomized study comparing Utrogestan® and Crinone® 8. Biomed Pap Med Fac Univ Palacky Olomouc Czech Repub.

[r15] Satir F, Toptas T, Inel M, Erman-Akar M, Taskin O (2013). Comparison of intravaginal progesterone gel and intramuscular 17-α-hydroxyprogesterone caproate in luteal phase support. Exp Ther Med.

[r16] Shapiro DB, Pappadakis JA, Ellsworth NM, Hait HI, Nagy ZP (2014). Progesterone replacement with vaginal gel versus i.m. injection: cycle and pregnancy outcomes in IVF patients receiving vitrified blastocysts. Hum Reprod.

[r17] Sofuoglu K, Gun I, Sahin S, Ozden O, Tosun O, Eroglu M (2015). Vaginal micronized progesterone capsule versus vaginal progesterone gel for lutheal support in normoresponder IVF/ICSI-ET cycles. Pak J Med Sci.

[r18] Tay PY, Lenton EA (2005). The impact of luteal supplement on pregnancy outcome following stimulated IVF cycles. Med J Malaysia.

[r19] Tournaye H, Sukhikh GT, Kahler E, Griesinger G (2017). A Phase III randomized controlled trial comparing the efficacy, safety and tolerability of oral dydrogesterone versus micronized vaginal progesterone for luteal support in in vitro fertilization. Hum Reprod.

[r20] Wang LJ, Huang FJ, Kung FT, Lin PY, Chang SY, Lan KC (2009). Comparison of the efficacy of two vaginal progesterone formulations, Crinone 8% gel and Utrogestan capsules, used for luteal support in blastocyst stage embryo transfers. Taiwan J Obstet Gynecol.

[r21] Zaman AY, Coskun S, Alsanie AA, Awartani KA (2017). Intramuscular progesterone (Gestone) versus vaginal progesterone suppository (Cyclogest) for luteal phase support in cysles of in vitro fertilization-embryo transfer: patient preference and drug efficacy. Fertil Res Pract.

